# Editorial

**DOI:** 10.1093/braincomms/fcz051

**Published:** 2020-01-13

**Authors:** Tara L Spires-Jones

**Affiliations:** Edinburgh, UK

Happy New Year and welcome to our second volume of *Brain Communications*.

Our first volume, launched in March 2019, was an adventure for the team and a fantastic start towards our goal to develop a journal that publishes rigorous studies in translational neuroscience. We were overwhelmed by the early success in terms of submissions to the journal with over 190 submissions within our first 9 months including over 100 transfers from our sister journal *Brain*.

**Figure 1:**
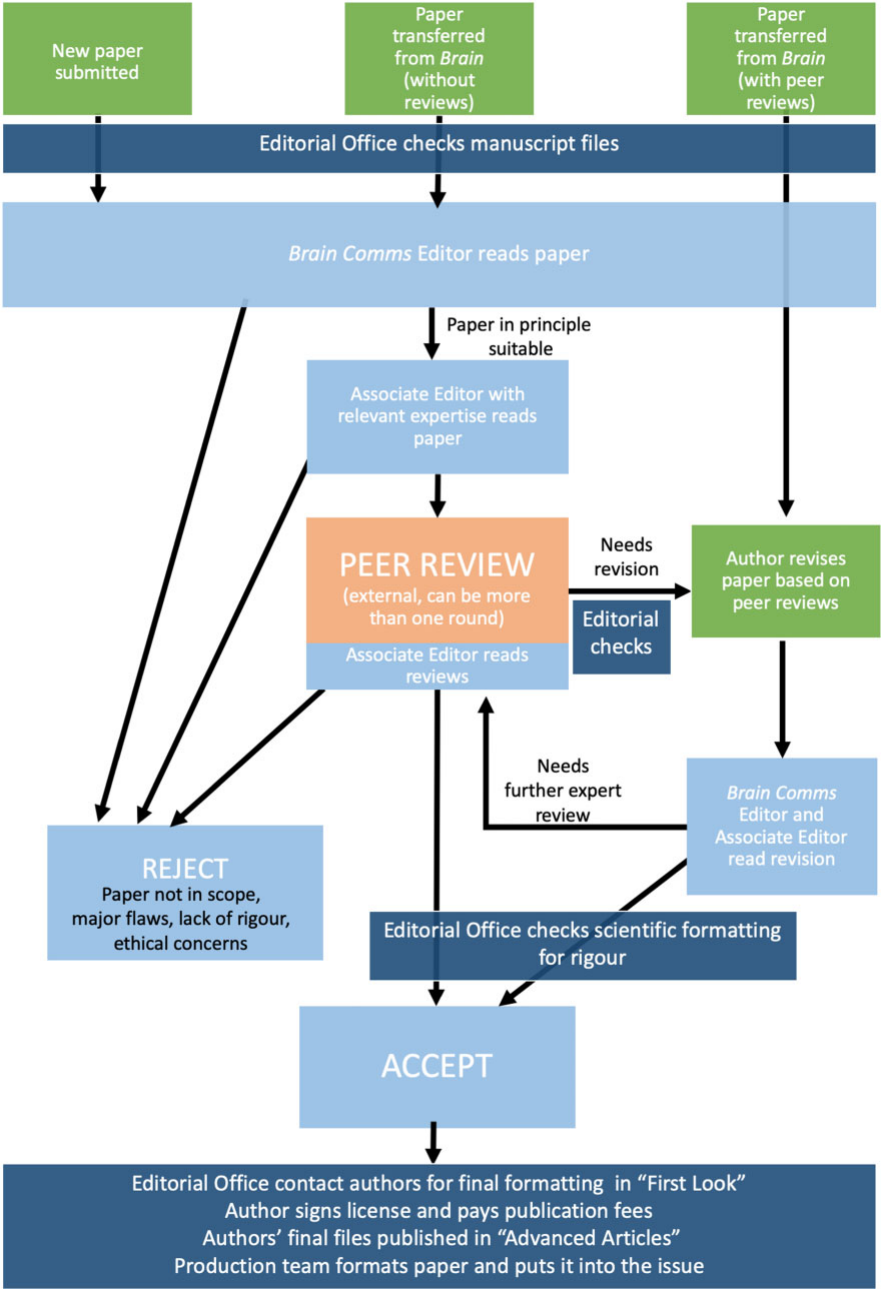
Flow chart of the journey of papers at Brain Communications.

In case you are interested in the publishing process at *Brain Communications*, I have made a flow chart of the journey of manuscripts we receive (Fig. 1). You will see that peer review is at the heart of our publishing process, and I want to thank our reviewers for their essential contributions to the journal. We offer authors and reviewers the choice to publish peer reviews alongside papers. If authors and all reviewers agree, we publish the reviews, which we hope sheds some light on the work put in by scientists all over the world in reviewing papers for journals like ours. One example of this is in the paper Mehta *et al.* (2019) where in the supplementary data, you can find the reviews and the authors’ responses. In Fig. 1, you can also see that our editorial team checks papers and adds feedback to authors to try to ensure consistently high level of robustness in figures and data analysis.

The papers we have published so far cover a broad range of topics in neurology, psychiatry and neuroscience from cell and animal models of diseases to human cohort studies. The new cover figure for volume 2 comes from Altmann *et al.* (2019) who have found that polygenic risk score excluding the *APOE4* locus associates with clinical diagnosis and biomarkers. The images show regional associations between amyloid PET tracer uptake and polygenic risk scores in the Alzheimer’s Disease Neuroimaging Initiative cohorts.

As well as covering a wide breadth of translational neuroscience subjects, our journal so far has submissions from authors in over 25 countries and many stages of career from undergraduate students to technicians to professors. *Brain Communications* papers have been well received with the first citations of our manuscripts appearing in other peer-reviewed journals including *Molecular Psychiatry* and *Cell Reports*.

We also have good coverage in traditional and social media outlets given the young age of our journal: more than 800 mentions tracked by Altmetrics.com (as of 17 December 2019). A popular paper for online news was Stuart Washington’s article on exercise in working memory in Gulf War Illness (Washington *et al.*, 2019).

We use our Twitter account @BrainComms to share news of recent papers and are happy to already have over 700 followers. Our papers are proving popular on Twitter. For example, Rimona Weil’s paper on neuroimaging findings implicating a hippocampal network in Parkinson’s disease dementia symptoms (Weil *et al.*, 2019) was tweeted 79 times. We are also grateful to this year’s most prolific Twitter follower Dervis Salih who has been very supportive in sharing our papers and also published work in *Brain Communications* suggesting that genetic variability in the microglial response to amyloid deposition is a major determinant for Alzheimer’s disease risk (Salih *et al.*, 2019).

All of the profits from the publication charges for our articles have contributed to the good works from the Guarantors of Brain [an UK Registered Charity (264139)] and our academic publishing partner Oxford University Press. The Guarantors of Brain support travel grants, fellowships, meetings and events to enhance public understanding of neurology, which you can apply for here: https://guarantorsofbrain.org/grants/. More about how Oxford’s share of any profits is returned to academia can be found here: https://www.ox.ac.uk/clarendon.

In the year to come, we hope to build up the journal to include ‘field potential’ articles as well as publishing more strong scientific papers. If you have any ideas for articles about promoting rigour in translational neuroscience or promoting career development of translational neuroscientists, please get in touch: brcoms.editorialoffice@oup.com.

Finally, thank you to our authors, reviewers, editorial team and readers for making our first issue of *Brain Communications* such a success. Wishing all of you a healthy, happy 2020.
